# Use of spatial panel-data models to investigate factors related to incidence of end-stage renal disease: a nationwide longitudinal study in Taiwan

**DOI:** 10.1186/s12889-023-15189-7

**Published:** 2023-02-06

**Authors:** Chien-Chou Su, Kuo-Jung Lee, Chi-Tai Yen, Lu-Hsuan Wu, Chien-Huei Huang, Meng-Zhan Lu, Ching-Lan Cheng

**Affiliations:** 1 Clinical Innovation and Research Center, National Cheng Kung University Hospital, Tainan City, Taiwan; 2 Department of Statistics, Institute of Data Science, National Cheng Kung University, No.1, University Road, 701 Tainan City, Taiwan; 3 Department of Nephrology , Tainan Hospital, Ministry of Health and Welfare , Tainan City, Taiwan; 4 Department of Pharmacy, National Cheng Kung University Hospital, Tainan City, Taiwan; 5 School of Pharmacy, Institute of Clinical Pharmacy and Pharmaceutical Sciences, National Cheng Kung University, No. 1 University Road, 701 Tainan city, Taiwan

**Keywords:** Spatial analysis, End-stage renal disease, Risk factor, Taiwan, NSAIDs, Aminoglycoside

## Abstract

**Background:**

The assumptions of conventional spatial models cannot estimate the responses across space and over time. Here we propose new spatial panel data models to investigate the association between the risk factors and incidence of end-stage renal disease (ESRD).

**Methods:**

A longitudinal (panel data) study was conducted using data from the National Health Insurance Database in Taiwan. We developed an algorithm to identify the patient’s residence and estimate the ESRD rate in each township. Corresponding covariates, including patient comorbidities, history of medication use, and socio-environmental factors, were collected. Local Indicators of Spatial Association were used to describe local spatial clustering around an individual location. Moreover, a spatial panel data model was proposed to investigate the association between ESRD incidence and risk factors.

**Results:**

In total, 73,995 patients with ESRD were included in this study. The western region had a higher proportion of high incidence rates than the eastern region. The proportion of high incidence rates in the eastern areas increased over the years. We found that most “social environmental factors,” except average income and air pollution (PM 2.5 and PM10), had a significant influence on the incidence rate of ESRD when considering spatial dependences of response and explanatory variables. Receiving non-steroidal anti-inflammatory drugs and aminoglycosides within 90 days prior to ESRD had a significant positive effect on the ESRD incidence rate.

**Conclusion:**

Future comprehensive studies on townships located in higher-risk clusters of ESRD will help in designing healthcare policies for suitable action.

**Supplementary Information:**

The online version contains supplementary material available at 10.1186/s12889-023-15189-7.

## Background

End-stage renal disease (ESRD) is highly prevalent globally and is recognized as a major public health problem worldwide with a substantial financial burden [1]. The strong correlation between geographic factors and the incidence of ESRD has previously been observed through spatial analysis [2]. Geographic factors include socioeconomic characteristics, medical resources, demographic structure, and environmental status. In addition to geographic factors, studies have shown that Asian ethnicity, hypertension or diabetes, and poor medical care are potential risk factors for ESRD [3]. In addition, exposure to nephrotoxic drugs, especially radiocontrast, non-steroidal anti-inflammatory drugs (NSAIDs), aminoglycosides, and renin-angiotensin-aldosterone system (RAAS) inhibitors can lead to acute kidney injury, which then progresses to ESRD [4]. However, the effect of these factors on ESRD development after adjusting for geographic factors remains unclear.

Conventional spatial models assume that responses across space and over time can be modelled using a single spatial regression model. However, such models are increasingly being questioned because of their unrealistic simplification of the response variables. In ESRD data, the ESRD incidence of a particular unit may behave in a particular manner depending on the nearby units, independent explanatory variables taken by other units, and on the interaction effects among the error terms for a spatial interaction process [2, 5–9]. Most previous studies investigated the association between end-stage renal disease (ESRD) incidence with person-level covariates or geographical variance, but none considered the dual effect of temporal and spatial factors on the occurrence of ESRD. In addition to geographical factors, changes in time may affect the incidence of ESRD. Therefore, we applied the spatial panel model to estimate the risk of ESRD in CKD patients associated with demographics, comorbidities, medications, social environmental, and socioeconomic factors when considering geographical and temporal factors.

Consequently, flexible and interpretable spatial panel data models [10] have often been utilized to explain three different types of interaction effects: (1) endogenous interaction risk effects among response variables, (2) endogenous interactions among explanatory variables, and (3) exogenous interaction risk effects and error terms correlated over different regions in space and time frames. In this study, we hypothesized that the incidence of ESRD is not only affected by personal risk factors (such as diseases and medications), but also by the interaction of spatial and temporal (geographical information) factors. To account for these interactions, we applied Spatial Panel Data Models (SPDMs) to investigate the risk factors related to the incidence of ESRD.

## Methods

### Data source

Data extracted from the National Health Insurance Database (NHID) was used in this study. The National Health Insurance (NHI) is a universal and mandatory healthcare insurance program that covers 99% of the Taiwanese population. Thus, the NHID contains representative and long-term follow-up data for epidemiological studies. The NHID contains a registry of beneficiaries, ambulatory care claims, inpatient claims, prescription dispensing claims of pharmacies, and a registry for catastrophic illness. Each claim has diagnostic codes according to the International Classification of Diseases, Ninth Edition, Clinical Modification (ICD-9-CM), and details on medications, including the date of prescription, days of supply, and National Health Insurance Drug Codes for all reimbursed pharmaceutical products. These datasets can be linked to personal identification numbers and can provide patient-level clinical information.

### Design, setting, and study population

**Fig. 1:**
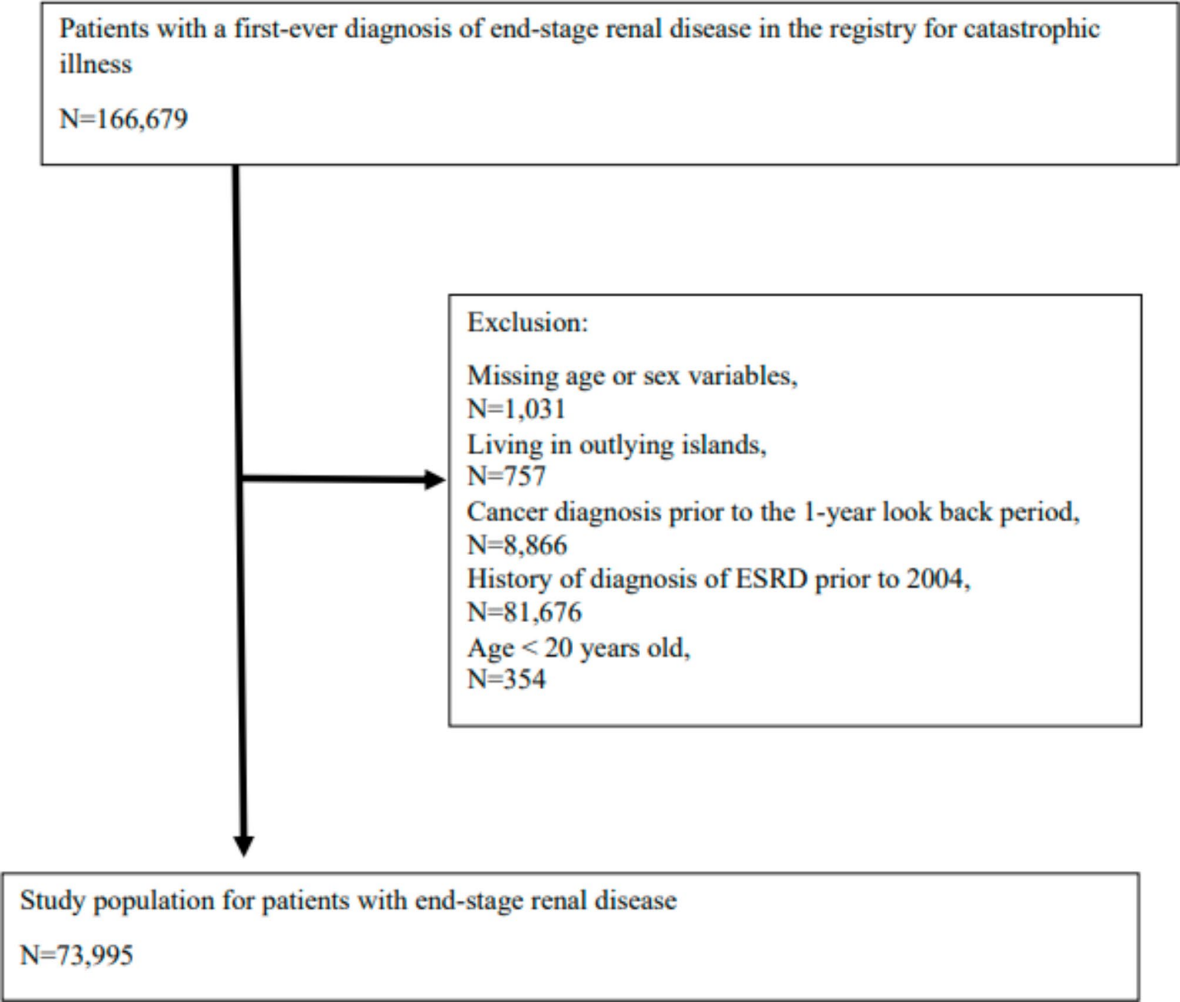
Assembly of the study population

We conducted a longitudinal (panel data) study. Patients were identified from the registry for catastrophic illness and diagnosed with ESRD (ICD-9-CM:585) between 2004 and 2011. The index date was defined as the registered date. Patients were excluded if they had missing information on age and sex variables, lived in outlying islands, were diagnosed with cancer during the 1-year look-back period prior to the index date, had a history of ESRD diagnosis prior to 2004, or were younger than 20 years old. The study population is shown in Fig. 1.

The patient’s residence was identified according to the algorithm of residence estimation developed by Lin et al. [11], and validated by census data (Pearson correlation coefficient = 0.960). The rule of residence estimation was based on the classification of beneficiaries in the NHI program and the location of hospital or clinic visits. This estimation was composed of four steps: (1) if the employment status of a beneficiary belongs to a certification requiring a professional license (such as accountant, lawyer, health care personnel), farmers, fishermen, veterans, or low-income citizens, whose residence can be designated as identical to the one registered in the NHI registry, as professional practice or veteran, and social benefit is restricted to the location of a city or county. The employment status can be identified from the database of beneficiary registry with the code numbers of “2 (62 prior to 2006),” “3,” “5,” or “6”; (2) for those who did not belong to the above categories, we retrieved the ambulatory care for upper respiratory tract infection (URI) in NHID, and assigned the location of hospital/clinic they visited as their residence area; (3) if the persons had no records for ambulatory URI, we checked for other ambulatory visits in the hospital/clinic and selected the one with highest visits as their residence location; (4) for those who had no claims record of ambulatory care other than the six defined chronic illnesses, the residence information in the most recent registry was considered to be their residence location. The residence estimation algorithm is shown in supplementary Figure S1. and Table S1, which shows the validated results of residence estimation positive predictive value (PPV) by linking NHID and the National Health Interview Survey (NHIS). The overall residence estimation PPV was 84.3%.

### Covariates

The patients’ comorbidities and history of medication use were recorded during a 1-year lookback period prior to the index date, including hypertension (ICD-9-CM:401–405), diabetes mellitus (ICD-9-CM:250), NSAIDs, aminoglycosides, and contrast media. The dosage of medication was converted into the defined daily doses (DDD), as defined by the World Health Organization (WHO), to measure the consumption of medications. The DDD of a pharmaceutical substance represents the assumed average maintenance dose per day of a drug when used as its main indication in adults. The medication utilization was defined as the DDD per day per 1,000 persons (DDD/day/1,000 persons).

The socio-environmental factors included the proportion of old adults, proportion of aboriginal people, proportion of healthcare resource allocation surrogates, proportion of bachelor’s degrees, unemployment rate, and average monthly income (NT$ 1,000) in each county in Taiwan. These data were obtained from the Open Data, Ministry of the Interior (https://data.gov.tw/). Information on particulate matter (PM2.5 and PM10) was obtained from the Environmental Protection Administration, Taiwan. The average PM2.5 and PM10 values were recorded per year in each county in this study.

### Statistical analysis

Descriptive statistics were used to summarize the baseline characteristics. Continuous variables were described as means with standard deviations (SD), and categorical variables were described as numbers and proportions [12]. The incidence of ESRD was defined as the number of new cases divided by the total number of residents in each township in each year multiplied by 100,000. The incidence rates were further stratified into six quantiles: ≤ 10, 10–20, 20–30, 30–40, 40–50, and > 50 per 100,000 persons.

The Local Indicators of Spatial Association (LISA) is a measure used to investigate significant local spatial clustering around an individual location [13]. If the LISA statistic testing is not significant, then no spatial pattern is present in the areas that indicate random spatial observations. When the LISA statistic testing is significant, there are two possible patterns of local spatial clusters to be exhibited: (1) the observation is higher than the average of the entire study area, and its neighbors indicate a high-high (HH) association, the so-called hot spot; (2) both the observation and its neighbors are lower than the average, and the spatial tendency is low-low (LL), the so-called cold spot.

To account for time-dependent and spatial clustering in the responses and covariates, a spatial panel data model, called Spatial Durbin Model (SDM) [14], was proposed to study the association between ESRD incidence and risk factors. The statistical model is given as:

$$\begin{array}{l}{Y_{it}} = \rho W{Y_{it}} + \lambda W{X_{it}} + \beta {X_{it}} + \mu ,i = 1, \ldots n;\\t = 1, \ldots ,T\end{array}$$

where ${Y}_{it}$ is the natural logarithm of the incidence rate of ESRD, ${X}_{it}$ is the corresponding risk factor at time $t$ in location $i$; ρ is the spatial lag coefficient, $\lambda$ is the spatial autoregressive effect, β is a vector of coefficients of explanatory variables, $W$ is the spatial weights matrix, and µ is the spatial autocorrelation of an error.

Spatial spillover refers to the impact of a health outcome in a target area affected by health outcomes in neighboring areas, or the impact of a health outcome in a target area that is affected by risk factors in neighboring areas [15]. Thus, ρ and $\lambda$ are relevant to spatial spillover effects. In the present study, multicollinearity was checked. We found PM2.5 and PM10, and aminoglycosides utilization in different days showed highest correlation. Therefore, we excluded the variables of PM10, aminoglycosides in 91–180 days and ≧ 180 days from spatial panel model which was based on the hypothesis. All significance levels were two-sided, with $p\text{-value} < 0.05$ indicating statistical significance. Statistical analyses were performed using SAS 9.4, Stata 13 (xsmle package), and ArcGIS 10.5 software.

## Results

**Fig. 2:**
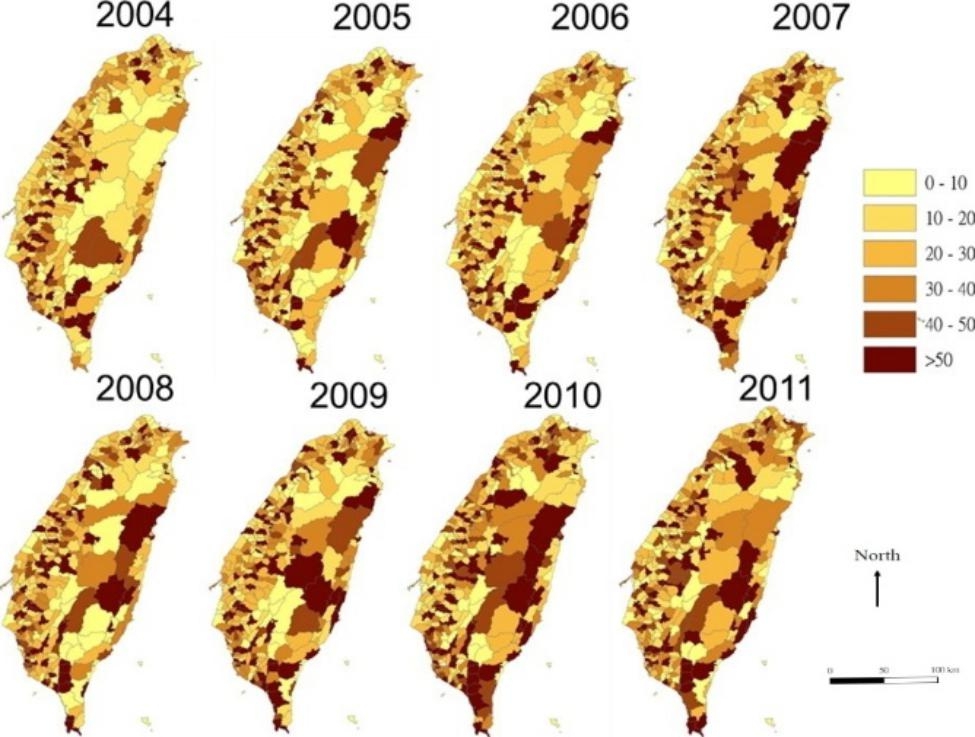
Incidence of end-stage renal disease in patients with chronic kidney disease from 2004–2011

In total, 73,995 patients with ESRD were included in this study. The mean age was 63.8 (SD 14.3) years old (Table 1). Overall, 60.4% and 90.6% patients with ESRD had diabetes mellitus and hypertension, respectively. These patients had a higher proportion of NSAIDs use (74.5%), and higher utilization of aminoglycoside within 90 days prior to ESRD (68.0 DDD/day/1,000 persons) and NSAIDs during 181–365 days prior to ESRD (795.3 DDD/day/1,000 persons). Figure 2 shows the distribution of ESRD incidence in different townships in Taiwan between 2004 and 2011. The darker area indicates a higher incidence. We found that there was a higher proportion of high incidence rates in the western region than in the eastern region. The proportion of high incidence rates in the eastern areas increased over the study period.

**Table 1:** Baseline characteristics of the study population

Variables	No. of patients	% (or SD)
*Total*	*73,995*	*100*
*Demographics*		
Age, mean, SD	64	14.32
Age, n, %		
< 45	7,264	9.8
45–64	28,685	38.8
65–74	18,915	25.6
> 75	19,131	25.9
Male, n, %	37,685	50.9
Calendar year, n, %		
2004	8,454	11.4
2005	9,000	12.2
2006	8,659	11.7
2007	9,077	12.3
2008	9,463	12.8
2009	9,543	12.9
2010	10,040	13.6
2011	9,759	13.2
*Comorbidity*		
Diabetes mellitus, n, %	44,695	60.4
Hypertension, n, %	67,053	90.6
*History of medication use*		
NSAIDs	55,119	74.5
Aminoglycosides	12,882	17.4
Contrast media	0	0
*Dosage of medication use (DDD/day/1,000 persons)*		
NSAIDs		
Within 90 days prior to ESRD	666.3	
91–180 days prior to ESRD	509.1	
181–365 days prior to ESRD	795.3	
Aminoglycoside		
Within 90 days prior to ESRD	68.0	
91–180 days prior to ESRD	20.9	
181–365 days prior to ESRD	22.9	
*Residence, n, %*		
North	30,160	40.8
Central	17,960	24.3
South	23,776	32.1
East	2,099	2.8

**Fig. 3:**
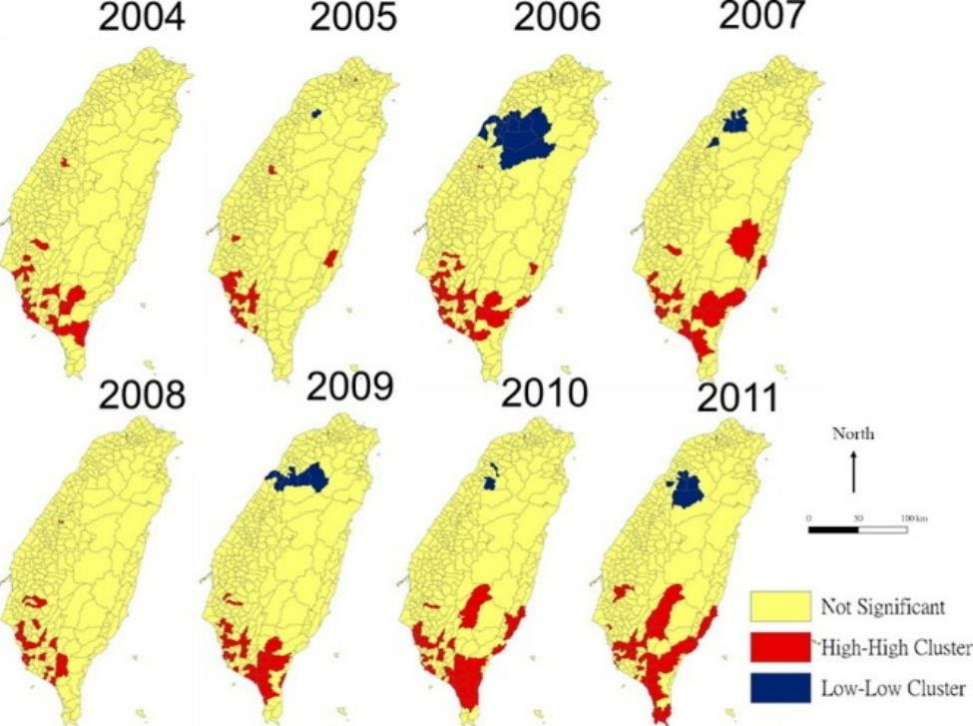
Local indicators of spatial association for the incidence of end-stage renal disease in patients with chronic kidney disease from 2004–2011

Figure 3 shows the spatial clustering of the ESRD incidence rate in different townships by year. Most of the high-high associations (hot spots) each year were in southern Taiwan, which indicated that the southern region had a higher-than-average ESRD incidence compared to other parts of Taiwan. A high-high association with the incidence of ESRD was found in the southern and southeast region cross years.

Three spatial panel data models were used with three different hierarchies to evaluate the risk factors for the incidence rate of ESRD: (1) socio-environmental factors (Model 1), (2) socio-environmental factors and comorbidities (Model 2), and (3) socio-environmental factors, comorbidities, and medications (Model 3). Model 3 is the full model, followed by the results of Model 3. Regarding socio-environmental factors, the proportion of older adults (coef. = 0.099, p < 0.001), aboriginal people (coef. = 0.0167, p < 0.001), healthcare resource allocation surrogates (coef. = 0.008, p = 0.025), and bachelor’s degrees (coef. = -0.0612, p < 0.001), as well as the unemployment rate (coef. = 0.0741, p < 0.001) were significantly associated with the ESRD incidence. Further, male gender (coef. =0.0124, p < 0.001), diabetes mellitus (coef. = 0.0015, p < 0.001), and hypertension (coef. = 0.0004, p < 0.001) all increased ESRD risk, as did NSAID use (coef. = 1.72, p = 0.007) (Table 2, Model 3).

**Table 2:** Spatial panel-data models of the incidence of end-stage renal disease in patients with chronic kidney disease

Variable	Model 1				Model 2				Model 3			
	Coef.	Std. Err.	z-value	p-value	Coef.	Std. Err.	z-value	p-value	Coef.	Std. Err.	z-value	p-value
*Main effects*												
*Social environmental & socioeconomic factors*												
Proportion of old adults (%)	0.0947	0.0111	8.55	< 0.001	0.1000	0.0088	11.35	< 0.001	0.0990	0.0095	10.38	< 0.001
Proportion of aboriginal peoples (%)	0.0231	0.0025	9.23	< 0.001	0.0171	0.0022	7.65	< 0.001	0.0167	0.0024	6.98	< 0.001
Proportion of healthcare resource allocation surrogate (%)	0.0137	0.0039	3.53	< 0.001	0.0076	0.0036	2.12	0.034	0.0080	0.0036	2.24	0.025
Proportion of bachelor’s degree (%)	-0.0536	0.0082	-6.57	< 0.001	-0.0596	0.0085	-7.03	< 0.001	-0.0612	0.0089	-6.84	< 0.001
Unemployment rate (%)	0.0782	0.0200	3.90	< 0.001	0.0689	0.0188	3.66	< 0.001	0.0741	0.0193	3.85	< 0.001
Average income per month (NT$ 1,000)	1.88E-07	2.36E-07	0.80	0.424	3.67E-07	1.99E-07	1.85	0.065	4.03E-07	2.03E-07	1.98	0.047
PM2.5 (µg/m3)	1.42E-05	1.48E-03	0.01	0.992	0.0009	0.0012	0.77	0.442	0.0009	0.0012	0.75	0.456
*Baseline characteristics*												
Proportion of male (%)					0.0125	0.0013	9.88	< 0.001	0.0124	0.0013	9.89	< 0.001
Average age (year)					0.0008	0.0010	0.84	0.402	0.0008	0.001	0.85	0.396
Diabetes mellitus (%)					0.0015	0.0003	5.18	< 0.001	0.0015	0.0003	5.07	< 0.001
Hypertension (%)					0.0073	0.0004	18.93	< 0.001	0.0072	0.0004	17.77	< 0.001
*NSAIDs or Aminoglycosides (DDD/person/day)*												
NSAID: ≦ 90 days prior to ESRD									1.7200	0.6433	2.67	0.007
NSAID: 91–180 days prior to ESRD									0.2823	0.6587	0.43	0.668
NSAID: 181–365 days prior to ESRD									-0.2832	0.4901	-0.58	0.563
AG: ≦ 90 days prior to ESRD									-0.2370	1.1646	-0.2	0.839
***Spatial spillover effect***												
Lambda (λ)	0.0860	0.0139	-4.33	< 0.001	0.0581	0.0228	2.55	0.011	0.0661	0.0221	3.00	0.003
Rho (ρ)	-0.0781	0.0180	6.18	< 0.001	-0.0388	0.0235	-1.65	0.099	-0.0465	0.0236	-1.97	0.049
***Goodness of fit***												
Log-likelihood ratio	-1118.0				-775.6				-772.0			

Note: NSAID: non-steroidal anti-inflammatory drug; AG: aminoglycosides

After the model estimation, we concluded the following relationships between the changes in the ESRD incidence rate and the explanatory variables in groups of risk factors in this study. All models reveal that most “social environmental and socioeconomic factors”, except average income and PM 2.5, exerted a significant influence on the incidence rate of ESRD when considering spatial dependences of response and explanatory variables. The proportion of older adults, aboriginal people, healthcare resources, and unemployment rate clearly had a positive impact on the ESRD incidence rate. We further found that the unemployment rate and proportion of older adults were stronger than others in the changes in the ESRD incidence rate. However, the proportion of bachelor’s degrees had a negative effect on the ESRD incidence rate. Implicitly, a high proportion of bachelor’s degrees would reduce ESRD incidence rate. Changes in the average monthly income, PM2.5, and PM10 did not affect the ESRD incidence rate. Among the group of risk factors for baseline characteristics, we found that males with diabetes and hypertension were more likely to have ESRD. In the use of NSAIDs or aminoglycosides, taking NSAIDs and aminoglycosides within 90 days prior to ESRD showed a weaker and significantly positive effect on the ESRD incidence rate; however, taking aminoglycosides 181–365 days prior to ESRD resulted in a significantly reduced ESRD incidence rate. This may indicate that ESRD occurrence is associated with recent rather than distant exposure.

The models we constructed revealed several spatial patterns in the ESRD incidence rate, as shown in Figs. 2 and 3. The positive spatial autoregressive coefficient, ${\uprho }$, indicates that the changes of ESRD incidence in the nearby townships behave similarly. That the spatial spillover effect, ${\uplambda }$, is not significant implies that it is the weak spatial dependence in the risk factors in the model (Table 2).

## Discussions

In this study, we collected personal and environmental information of patients with ESRD across the years to investigate the spatial and temporal association between risk factors and ESRD incidence. In the spatial analysis, we identified spatial clustering effects for ESRD incidence rate, and a high-high association (hot spot) was observed in southern Taiwan every year.

Tainan City, Kaohsiung City, and Pingtung County were indicated as cities with spatial correlations for eight consecutive years were. In Pingtung County, we considered the possible reason was insufficient healthcare resources resulting in higher incidences of ESRD than average. A previous study indicated that a high incidence of ESRD could be attributed to the quality of health care and medical human power [16]. Thus, it is likely that the healthcare resources in this area were insufficient. In contrast to Pingtung County, Tainan City and Kaohsiung City are major cities in Taiwan. Although they have sufficient healthcare resources, they still showed relatively high incidences of ESRD in this study. It is worth noting that Pingtung County is located near Kaohsiung City, and therefore geographic factors may play a role in the high incidence; for example, a spatial spillover effect could affect the incidence of ESRD in Kaohsiung City. It was for this reason that we used spatial panel data models to investigate the association with the incidence of ESRD.

Considering time-dependent and spatial effects, we applied a spatial panel data model to estimate the marginal effects of personal and environmental factors on the incidence of ESRD. We found that environmental factors, such as population composition (proportion of old adults and level of education), healthcare resources, and unemployment rate, were associated with the incidence of ESRD. Personal risk factors, including diabetes, hypertension, NSAIDs, and aminoglycosides, were independent risk factors for ESRD after adjusting for spatial and temporal effects. Additionally, we used the diagnosis of hypertension and diabetes mellitus as covariates which were important risk factors for ESRD occurrence. Further, the Charlson Comorbidity Index (CCI) should be considered as a morbidity score if mortality is set as a dependent variable. Furthermore, the spatial spillover effect was not significant, implying that personal and local environmental factors were more substantial than the effects in neighboring areas after adjusting for all covariates.

The residential location in the registry of the NHID is not always equal to the residential area. Therefore, we applied the algorithm of residence area proposed by Lin et al. [17], and linked the NHID to the NHIS to obtain the actual residence area and evaluated the accuracy using this algorithm. The average PPV was approximately 84.3%, suggesting that the algorithm of the residence area is suitable to estimate a person’s residence area in the NHID registry.

The spatial analysis showed a high clustering effect of ESRD incidence in the southern and southeastern townships. These townships are the most remote areas with insufficient healthcare resources, such as the number of hospitals, medical staff, and beds, compared to the northern areas in Taiwan. A previous study indicated that different hospital sizes and medical manpower resources were associated with the deterioration of renal function [8]. This may be one of the reasons for the higher ESRD incidence in remote areas. There were several urban townships in the southern areas, such as Tainan City and Kaohsiung City which also showed a high clustering effect of ESRD incidence, even though these areas have sufficient healthcare resources. The reason for the higher ESRD incidence is different from that in other remote areas. A previous study reported that sufficient healthcare resources could improve accessibility. For example, patients would be more likely to accept dialysis because they were able to access healthcare resources [16].

Spatial analysis is a univariate analysis that cannot be used to investigate the association between multiple factors and outcomes. Therefore, a spatial panel data model can be used to evaluate the effect of a risk factor after adjusting for spatial and time-dependent effects and other risk factors. In the spatial panel-data model analysis, a higher proportion of older adults would increase the risk of ESRD, which was similar to a previous study in which renal function was found to progress with age [18]. A study in the United States of America reported that there was a higher rate of rapid deterioration of renal function in towns with a high proportion of ethnic minorities or immigrants (OR = 1.25, 95% Cl = 1.2–1.31) [16], and this finding was similar to our result that a higher proportion of aboriginal people was associated with a higher rate of ESRD incidence. Most aboriginal people lived in remote townships in Taiwan, which had insufficient healthcare resources, and were associated with an increased ESRD risk. The area with a higher proportion of bachelor’s degree was significantly associated with a lower ESRD incidence, which agrees with a previous study that found that the higher the level of education, the lower the incidence of diseases and deaths from diseases [16, 18]. People with higher education are more likely to realize and accept health suggestions [19], have a higher socioeconomic status, and be able to afford medical expenses [20]. We also found that a higher unemployment rate was associated with an increased risk of ESRD, which could be attributed to socioeconomic status [20].

NSAIDs and aminoglycosides are well-known nephrotoxic agents. However, in clinical observational studies, the detrimental effect of NSAIDs in chronic kidney disease (CKD) progression was inconsistent. In a prospective observational study of 10,184 individuals older than 65 years, the increased risk of CKD progression was only seen in NSAIDs users with glomerular filtration rate (GFR) > 60 mL/min/1.73 m2, but not in NSAID users with GFR < 60 mL/min/1.73 m2. This study could not exclude the possible bias that, in clinical situations, greater NSAIDs use may be avoidable in patients with less renal function [21, 22]. In another prospective cohort study of 4,101 patients with rheumatoid arthritis, a significant deleterious effect of renal function decline was only seen in patients with CKD stage 4–5, but not in patients with CKD stage 1–3 [23]. In our study, through spatial analysis, we found that the use of NSAIDs or aminoglycosides within 90 days prior to the index day increased the incidence of ESRD. Therefore, we reinforced the importance of avoiding exposure to nephrotoxic agents in patients with CKD. Interestingly, the use of aminoglycoside within 180–365 days prior to the index date exerted protective effects against renal function decline in ESRD. The reason for this was not clear, and it may imply that early recognition of CKD and knowledge of further avoiding exposure to nephrotoxic agents are practical strategies to preserve renal function in patients with CKD.

Ignoring the spatial dependence on the response and explanatory variables may reduce the efficiency of the results, and produce biased and inconsistent model estimations. In this study, we considered spatial panel models to study the association of risk factors with ESRD, which include lags of the dependent variable and of the independent variables in both space and time, providing a useful tool to quantify the magnitude of effects from the variables, in both the short- and long-term. In the model, the direct effects of the variable on the dependent variable could be investigated in its own ESRD incidence rate, rather than the coefficient estimate of that variable. In addition, the indirect effects of spatial spillovers exist rather than the coefficient estimate of the spatially lagged dependent variable, and/or the coefficient estimates of the spatially lagged independent variables can be examined. We applied the models to empirical data and ESRD incidence rate data from townships in Taiwan from 2004 to 2011. In this model, we found that NSAIDs or aminoglycosides seem to have an upward and then downward significant effects on ESRD incidence rates (Table 2).

### Limitations and strengths

This study had several limitations. First, the patient’s residence may have been misclassified because of the uniform insurance system. Patients may undergo dialysis or have medical visits away from their place of residence. Our validation study showed that the lowest PPV was for Kinmen, an offshore island with limited healthcare resources. Second, the extraction of information on particulate matter (PM2.5 and PM10) from county monitoring stations, rather than townships, may result in some loss of precision concerning intra-township variability risk. Another limitation was the absence of data on renal function in patients who received NSAIDs or aminoglycosides. The risk of nephrotoxicity due to NSAIDs or aminoglycosides occurs in patients with an underlying renal disease.

The primary strength of this study was that it examined a confirmed ESRD population with minimal financial barriers and readily accessible healthcare under Taiwan’s full-coverage NHI program. We further estimated the risk factors for ESRD after adjusting for geographic influence, socio-environmental factors, comorbidities, and medications. This will provide useful information for policymaking in different regions by targeting potential risk factors.

Spatial panel data models provide a useful tool for capturing time-dependent and spatial clustering in responses and covariates. However, the specification of spatial weights matrix *W* and m was associated with a significant issue. Consequently, empirical studies follow a statistical approach driven by data analysis and are limited to one or a few pre-specified *W* matrices. Often, spatial weight matrices are specified in terms of a well-founded background for certain spatial interaction effects and the frequency of their use, resulting in a criticism in empirical studies. In view of this, it should be clear that the way of thinking and model selection strategies are used in most empirical studies to determine the structure of the spatial weights’ matrix *W.*

## Conclusion

The spatial panel data model is suitable to identify high-risk areas and risk factors for ESRD. The results obtained with this method highlight that nephrotoxic drugs are a major concern, and that health authorities should design relevant actions or education programs to improve the quality of care.

## Electronic supplementary material

Below is the link to the electronic supplementary material.

Supplementary Material 1

Supplementary Material 2

Supplementary Material 3

Supplementary Material 4

Supplementary Material 5

## Data Availability

The source of data for this research is the Taiwan NHID, which is second-hand data, only allowed to be used by Taiwanese, and can only be assessed onsite. For legal reasons, the raw/processed data required to reproduce the above findings cannot be shared at this time. Website: https://dep.mohw.gov.tw/dos/lp-2506-113.html. To obtain the data used in this study, please contact the Department of Statistics, Ministry of Health and Welfare, Taiwan (TEL: +886-2-8590-6811). Contact person: Ms. Chang (TEL: +886-2-8590-6818; email: styun@mohw.gov.tw).

## Listof abbreviations

ESRD: End-stage renal disease
NSAIDs: Non-steroidal anti-inflammatory drugs
RAAS inhibitors: Renin-angiotensin-aldosterone system
NHID: National Health Insurance Database
ICD: International Classification of Diseases
NHI: National Health Insurance
ICD-9-CM: Ninth Edition, Clinical Modification
URI: Upper respiratory tract infection
PPV: Positive predictive value,
NHIS: National Health Interview Survey
DDD: Defined daily doses
PM: Particulate matter
SD: Standard deviations
LISA: Local Indicators of Spatial Association
CKD: Chronic kidney disease
GFR: Glomerular filtration rate
