# Supplementary material for: Use of spatial panel-data models to investigate factors related to incidence of end-stage renal disease: a nationwide longitudinal study in Taiwan
Source: BMC Public Health. 2023 Feb 6;23:247. doi: 10.1186/s12889-023-15189-7 (PMC9901115; doi:10.1186/s12889-023-15189-7)
Supplement: Supplementary file 1 — Supplementary Material 1 [file 12889_2023_15189_MOESM1_ESM.docx]

Figure S1 The algorithm for residence estimation in the National Health Insurance Database

No

No

No

Yes

Yes

Yes

Yes

Patient cohort in National Health Insurance Database

The residence located in the registry

= residential area

4. No other record of outpatient visit

Location of the hospital/clinic

= residential area

3. Hospital/clinic with the highest outpatient visits

Location of the hospital/clinic

= residential area

2. Record of an outpatient visit for URI (ICD-9 code: 460-466, 480-487)

The residence location in the registry

= residence area

1. Employment status classification is “2” (or “62” before 2006), “3,” “5,” or “6”

Identification of a cohort of patients with chronic kidney disease from 2004–2012
