# Supplementary material for: Use of spatial panel-data models to investigate factors related to incidence of end-stage renal disease: a nationwide longitudinal study in Taiwan
Source: BMC Public Health. 2023 Feb 6;23:247. doi: 10.1186/s12889-023-15189-7 (PMC9901115; doi:10.1186/s12889-023-15189-7)
Supplement: Supplementary file 2 — Supplementary Material 2 [file 12889_2023_15189_MOESM2_ESM.docx]

Table S1. Positive predictive value for residence estimation in the National Health Insurance Database compared to the National Health Interview Survey

| N=3,384 | |
| --- | --- |
| County | Positive predictive value (PPV) |
| *Overall* | 84.3 |
| Yilan | 96.2 |
| Changhua | 94.9 |
| Taichung | 94.6 |
| Tainan | 93.1 |
| Taitung | 91.7 |
| Kaohsiung | 91.6 |
| Taoyuan | 90.3 |
| Pingtung | 89.4 |
| Miaoli | 89.3 |
| Nantou | 88.7 |
| Keelung | 88.3 |
| Penghu | 87.7 |
| Yunlin | 87.7 |
| Hualien | 87.2 |
| Taipei | 85.8 |
| Hsinchu | 77.8 |
| New Taipei | 77.2 |
| Chiayi | 75.0 |
| Kinmen | 66.2 |
