# Supplementary material for: Use of spatial panel-data models to investigate factors related to incidence of end-stage renal disease: a nationwide longitudinal study in Taiwan
Source: BMC Public Health. 2023 Feb 6;23:247. doi: 10.1186/s12889-023-15189-7 (PMC9901115; doi:10.1186/s12889-023-15189-7)
Supplement: Supplementary file 3 — Supplementary Material 3 [file 12889_2023_15189_MOESM3_ESM.docx]

Table S3. Panel data models (mixed effects models) for the incidence of end-stage renal disease in patients with chronic kidney disease.

| **Variable** | **Model 1** | | | | **Model 2** | | | | **Model 3** | | | |
| --- | --- | --- | --- | --- | --- | --- | --- | --- | --- | --- | --- | --- |
|  | **Coef.** | **Std. Err.** | **z-value** | **p-value** | **Coef.** | **Std. Err.** | **z-value** | **p-value** | **Coef.** | **Std. Err.** | **z-value** | **p-value** |
| *Social environmental & socioeconomic factors* |  |  |  |  |  |  |  |  |  |  |  |  |
| Proportion of old adults (%) | 0.1096 | 0.0087 | 12.63 | <0.001 | 0.1025 | 0.0078 | 13.06 | <0.001 | 0.0327 | 0.0046 | 7.06 | <0.001 |
| Proportion of aboriginal peoples (%) | 0.0279 | 0.0019 | 14.62 | <0.001 | 0.0194 | 0.0017 | 11.14 | <0.001 | 0.0102 | 0.0009 | 11.72 | <0.001 |
| Proportion of healthcare resource  allocation surrogate (%) | 0.0107 | 0.0044 | 2.44 | 0.015 | 0.0062 | 0.0039 | 1.58 | 0.115 | 0.0247 | 0.0024 | 10.43 | <0.001 |
| Proportion of bachelor's degree (%) | -0.0313 | 0.0047 | -6.66 | <0.001 | -0.0458 | 0.0045 | -10.27 | <0.001 | -0.0135 | 0.0032 | -4.2 | <0.001 |
| Unemployment rate (%) | 0.0435 | 0.0133 | 3.26 | 0.001 | 0.0451 | 0.0118 | 3.81 | <0.001 | 0.0307 | 0.0117 | 2.64 | 0.008 |
| Average income per month  (NT$ 1,000) | 1.13E-07 | 1.87E-07 | 0.6 | 0.546 | 2.47E-07 | 1.66E-07 | 1.49 | 0.137 | 1.51E-07 | 1.14E-07 | 1.33 | 0.183 |
| PM2.5 (μg/m3) | 0.0005 | 0.0012 | 0.43 | 0.664 | 0.0005 | 0.0011 | 0.51 | 0.609 | 0.0017 | 0.0010 | 1.67 | 0.095 |
| *Baseline characteristics* |  |  |  |  |  |  |  |  |  |  |  |  |
| Proportion of males (%) |  |  |  |  | 0.0122 | 0.0014 | 8.88 | <0.001 | 0.0118 | 0.0010 | 11.87 | <0.001 |
| Average age (year) |  |  |  |  | 0.0011 | 0.0011 | 1.05 | 0.292 | 4.89E-05 | 0.0011 | 0.05 | 0.964 |
| Diabetes mellitus (%) |  |  |  |  | 0.0015 | 0.0003 | 4.58 | <0.001 | 0.0014 | 0.0003 | 4.11 | <0.001 |
| Hypertension (%) |  |  |  |  | 0.0075 | 0.0004 | 19.66 | <0.001 | 0.0081 | 0.0004 | 20.73 | <0.001 |
| *NSAIDs or Aminoglycosides (DDD/person/day)* |  |  |  |  |  |  |  |  |  |  |  |  |
| NSAID: ≦ 90 days prior to ESRD |  |  |  |  |  |  |  |  | 1.6784 | 0.7290 | 2.3 | 0.021 |
| NSAID: 91-180 days prior to ESRD |  |  |  |  |  |  |  |  | 0.1745 | 0.7488 | 0.23 | 0.816 |
| NSAID: 181-365 days prior to ESRD |  |  |  |  |  |  |  |  | -0.0113 | 0.5458 | -0.02 | 0.983 |
| AG: ≦ 90 days prior to ESRD |  |  |  |  |  |  |  |  | -0.0834 | 1.3117 | -0.06 | 0.949 |
