# Supplementary material for: Use of spatial panel-data models to investigate factors related to incidence of end-stage renal disease: a nationwide longitudinal study in Taiwan
Source: BMC Public Health. 2023 Feb 6;23:247. doi: 10.1186/s12889-023-15189-7 (PMC9901115; doi:10.1186/s12889-023-15189-7)
Supplement: Supplementary file 4 — Supplementary Material 4 [file 12889_2023_15189_MOESM4_ESM.docx]

Table S4. Cities with a high-high (HH) association over 8 consecutive years.

| Name of City |
| --- |
| East District, Tainan City |
| Sinsing District, Kaohsiung City |
| Sanmin District, Kaohsiung City |
| Niaosong District, Kaohsiung City |
| Zuoying District, Kaohsiung City |
| Gangshan District, Kaohsiung City |
| Lingya District, Kaohsiung City |
| Pingtung County |
