# Supplementary material for: Use of spatial panel-data models to investigate factors related to incidence of end-stage renal disease: a nationwide longitudinal study in Taiwan
Source: BMC Public Health. 2023 Feb 6;23:247. doi: 10.1186/s12889-023-15189-7 (PMC9901115; doi:10.1186/s12889-023-15189-7)
Supplement: Supplementary file 5 — Supplementary Material 5 [file 12889_2023_15189_MOESM5_ESM.docx]

Table S2. Overview of the correlation matrix between variables.

|  | Mean age | DM (%) | HTN (%) | NSAIDs in 90 | NSAIDs in 180 | NSAIDs in 365 | AGs in 90 | AGs in 180 | AGs in 365 | Elder | aborigine | Health source | Education level | Unemployment rate | Income | PM2.5 | PM10 | Male (%) |
| --- | --- | --- | --- | --- | --- | --- | --- | --- | --- | --- | --- | --- | --- | --- | --- | --- | --- | --- |
| Mean age | 1 |  |  |  |  |  |  |  |  |  |  |  |  |  |  |  |  |  |
|  |  |  |  |  |  |  |  |  |  |  |  |  |  |  |  |  |  |  |
| DM (%) | R:0.100 | 1 |  |  |  |  |  |  |  |  |  |  |  |  |  |  |  |  |
|  | P<.0001 |  |  |  |  |  |  |  |  |  |  |  |  |  |  |  |  |  |
| HTN (%) | R:0.132 | R:0.524 | 1 |  |  |  |  |  |  |  |  |  |  |  |  |  |  |  |
|  | P<.0001 | P<.0001 |  |  |  |  |  |  |  |  |  |  |  |  |  |  |  |  |
| NSAIDs in 90 | R:-0.028 | R:0.119 | R:0.172 | 1 |  |  |  |  |  |  |  |  |  |  |  |  |  |  |
|  | P:0.139 | P<.0001 | P<.0001 |  |  |  |  |  |  |  |  |  |  |  |  |  |  |  |
| NSAIDs in 180 | R:0.049 | R:0.091 | R:0.135 | R:0.117 | 1 |  |  |  |  |  |  |  |  |  |  |  |  |  |
|  | P:0.01 | P<.0001 | P<.0001 | P<.0001 |  |  |  |  |  |  |  |  |  |  |  |  |  |  |
| NSAIDs in 365 | R:0.04 | R:0.087 | R:0.156 | R:0.179 | R:0.382 | 1 |  |  |  |  |  |  |  |  |  |  |  |  |
|  | P:0.026 | P<.0001 | P<.0001 | P<.0001 | P<.0001 |  |  |  |  |  |  |  |  |  |  |  |  |  |
| AGs in 90 | R:-0.043 | R:-0.023 | R:-0.069 | R:0.067 | R:0.059 | R:0.003 | 1 |  |  |  |  |  |  |  |  |  |  |  |
|  | P:0.022 | P:0.219 | P<.0001 | P:.0004 | P:0.002 | P:0.858 |  |  |  |  |  |  |  |  |  |  |  |  |
| AGs in 180 | R:-0.037 | R:-0.011 | R:-0.048 | R:0.027 | R:0.07 | R:-0.005 | R:0.748 | 1 |  |  |  |  |  |  |  |  |  |  |
|  | P:0.048 | P:0.573 | P:0.01 | P:0.148 | P:.0002 | P:0.803 | P<.0001 |  |  |  |  |  |  |  |  |  |  |  |
| AGs in 365 | R:-0.033 | R:-0.014 | R:-0.047 | R:0.052 | R:0.066 | R:0.031 | R:0.852 | R:0.778 | 1 |  |  |  |  |  |  |  |  |  |
|  | P:0.081 | P:0.471 | P:0.013 | P:0.006 | P:.0004 | P:0.098 | P<.0001 | P<.0001 |  |  |  |  |  |  |  |  |  |  |
| Elder | R:0.132 | R:-0.037 | R:-0.091 | R:-0.064 | R:0.005 | R:-0.006 | R:-0.045 | R:-0.035 | R:-0.019 | 1 |  |  |  |  |  |  |  |  |
|  | P<.0001 | P:0.051 | P<.0001 | P:.0008 | P:0.776 | P:0.744 | P:0.019 | P:0.068 | P:0.304 |  |  |  |  |  |  |  |  |  |
| Aborigine | R:-0.264 | R:-0.028 | R:-0.105 | R:0.049 | R:0.01 | R:0.03 | R:0.065 | R:0.051 | R:0.052 | R:-0.164 | 1 |  |  |  |  |  |  |  |
|  | P<.0001 | P:0.133 | P<.0001 | P:0.01 | P:0.613 | P:0.11 | P:.0006 | P:0.007 | P:0.006 | P<.0001 |  |  |  |  |  |  |  |  |
| Health source | R;0.088 | R:0.039 | R:0.067 | R:-0.039 | R:-0.03 | R:-0.033 | R:-0.005 | R:-0.013 | R:-0.007 | R:-0.141 | R:-0.139 | 1 |  |  |  |  |  |  |
|  | P<.0001 | P:0.04 | P:.0004 | P:0.038 | P:0.111 | P:0.084 | P:0.787 | P:0.504 | P:0.725 | P<.0001 | P<.0001 |  |  |  |  |  |  |  |
| Education level | R:0.128 | R:0.046 | R:0.125 | R:-0.074 | R:-0.064 | R:-0.085 | R:-0.031 | R:-0.029 | R:-0.031 | R:-0.33 | R:-0.398 | R:0.326 | 1 |  |  |  |  |  |
|  | P<.0001 | P:0.014 | P<.0001 | P<.0001 | P:.0008 | P<.0001 | P:0.1 | P:0.123 | P:0.1 | P<.0001 | P<.0001 | P<.0001 |  |  |  |  |  |  |
| Unemployment rate | R:0.041 | R:0.071 | R:0.044 | R:-0.07 | R:-0.039 | R:-0.012 | R:-0.006 | R:-0.012 | R:-0.008 | R:0.030 | R:0.022 | R:0.020 | R:0.077 | 1 |  |  |  |  |
|  | P:0.029 | P:.0002 | P:0.019 | P:.0002 | P:0.040 | P:0.517 | P:0.761 | P:0.511 | P:0.669 | P:0.108 | P:0.238 | P:0.282 | P<.0001 |  |  |  |  |  |
| Income | R:-.0002 | R:-0.023 | R:0.006 | R:-0.022 | R:-0.053 | R:-0.043 | R:-0.017 | R:-0.019 | R:-0.029 | R:-0.256 | R:-0.182 | R:0.079 | R:0.628 | R:-0.086 | 1 |  |  |  |
|  | P:0.992 | P:0.225 | P:0.771 | P:0.243 | P:0.005 | P:0.024 | P:0.370 | P:0.307 | P:0.127 | P<.0001 | P<.0001 | P<.0001 | P<.0001 | P<.0001 |  |  |  |  |
| PM_2.5_ | R:0.038 | R:0.022 | R:0.037 | R:-0.07 | R:-0.065 | R:-0.119 | R:-0.033 | R:-0.019 | R:-0.037 | R:-0.002 | R:-0.2 | R:0.034 | R:0.089 | R:-0.029 | R:-0.110 | 1 |  |  |
|  | P:0.048 | P:0.251 | P:0.054 | P:.0002 | P:.0006 | P<.0001 | P:0.084 | P:0.305 | P:0.048 | P:0.904 | P<.0001 | P:0.072 | P<.0001 | P:0.122 | P<.0001 |  |  |  |
| PM_10_ | R:0.005 | R:0.008 | R:0.051 | R:-0.006 | R:0.022 | R:-0.019 | R:0.014 | R:0.001 | R:-0.012 | R:-0.006 | R:-0.242 | R:0.032 | R:-0.017 | R:-0.036 | R:-0.218 | R:0.721 | 1 |  |
|  | P:0.794 | P:0.687 | P:0.007 | P:0.768 | P:0.247 | P:0.32 | P:0.446 | P:0.956 | P:0.526 | P:0.736 | P<.0001 | P:0.088 | P:0.374 | P:0.056 | P<.0001 | P<.0001 |  |  |
| Male (%) | R:-0.106 | R:0.05 | R:0.069 | R:0.004 | R:-0.036 | R:-0.013 | R:-0.04 | R:-0.024 | R:-0.039 | R:0.047 | R:0.012 | R:-0.001 | R:-0.013 | R:-0.005 | R:0.017 | R:0.017 | R:0.014 | 1 |
|  | P<.0001 | P:0.011 | P:.0004 | P:0.831 | P:0.062 | P:0.51 | P:0.04 | P:0.22 | P:0.046 | P:0.015 | P:0.522 | P:0.953 | P:0.5 | P:0.783 | P:0.389 | P:0.383 | P:0.484 |  |
